# *Pseudomonas aeruginosa* gene PA4880 encodes a Dps-like protein with a Dps fold, bacterioferritin-type ferroxidase centers, and endonuclease activity

**DOI:** 10.3389/fmolb.2024.1390745

**Published:** 2024-05-22

**Authors:** Nimesha Rajapaksha, Huili Yao, Aisha Cook, Steve Seibold, Lijun Liu, Kevin P. Battaile, Leo Fontenot, Fabrizio Donnarumma, Scott Lovell, Mario Rivera

**Affiliations:** ^1^ Department of Chemistry, Louisiana State University, Baton Rouge, LA, United States; ^2^ Protein Structure & X-ray Crystallography Laboratory, University of Kansas, Lawrence, KS, United States; ^3^ NYX, New York Structural Biology Center, Upton, NY, United States

**Keywords:** Dps, mini ferritin, iron homeostasis, iron metabolism, bacterioferritin, endonuclease, oxidative stress

## Abstract

We report the biochemical, structural, and functional characterization of the protein coded by gene PA4880 in the *P. aeruginosa* PAO1 genome. The PA4880 gene had been annotated as coding a probable bacterioferritin. Our structural work shows that the product of gene PA4880 is a protein that adopts the Dps subunit fold, which oligomerizes into a 12-mer quaternary structure. Unlike Dps, however, the ferroxidase di-iron centers and iron coordinating ligands are buried within each subunit, in a manner identical to that observed in the ferroxidase center of *P. aeruginosa* bacterioferritin. Since these structural characteristics correspond to Dps-like proteins, we term the protein as *P. aeruginosa* Dps-like, or Pa DpsL. The ferroxidase centers in Pa DpsL catalyze the oxidation of Fe^2+^ utilizing O_2_ or H_2_O_2_ as oxidant, and the resultant Fe^3+^ is compartmentalized in the interior cavity. Interestingly, incubating Pa DpsL with plasmid DNA results in efficient nicking of the DNA and at higher concentrations of Pa DpsL the DNA is linearized and eventually degraded. The nickase and endonuclease activities suggest that Pa DpsL, in addition to participating in the defense of *P. aeruginosa* cells against iron-induced toxicity, may also participate in the innate immune mechanisms consisting of restriction endonucleases and cognate methyl transferases.

## 1 Introduction

*Pseudomonas aeruginosa* is a Gram-negative highly adaptable opportunistic pathogen, which is often present in environments influenced by human activity (Crone et al., 2020). *P. aeruginosa* can cause acute and chronic infections in surgical incisions, and in patients with burn wounds, catheters, and diabetic foot ulcers (Burrows, 2018; Horcajada et al., 2019). Our laboratories have been studying the structure and function of iron storage proteins in *P. aeruginosa*. Ferritin and ferritin-like molecules are particular to iron storage because these molecules catalyze the oxidation of Fe^2+^ and compartmentalize the resultant Fe^3+^ in their interior cavity. Consequently, in addition to accumulating iron at concentrations much higher than those enabled by the poor solubility of Fe^3+^near neutral pH, ferritin and ferritin-like molecules minimize the toxic effects expected from unregulated Fe^2+^/Fe^3+^ redox cycling (Bradley et al., 2016; Rivera, 2017; Rivera, 2023). The *P. aeruginosa* PAO1 genome contains four genes encoding iron storage proteins: *ftnA* (PA4235) encodes a bacterial ferritin (FtnA) (Yao et al., 2011), *bfrB* (PA3531) codes for a bacterioferritin (BfrB) (Weeratunga et al., 2009; Weeratunga et al., 2010), gene PA0962 codes a Dps (Rajapaksha et al., 2023), and gene PA4880, the subject of this report, is annotated to encode a probable bacterioferritin. Understanding the function of all these proteins is an important step toward gaining a more comprehensive understanding of the fate of iron in bacterial cells, and how storage molecules contribute to managing the benefits and risks associated with the chemistry of essential iron.

Our investigations have recently demonstrated that *P. aeruginosa* stores iron in a heteropolymeric bacterioferritin (Bfr) composed from two distinct types of subunits, a bacterial ferritin, FtnA coded by gene PA4235, and a bacterioferritin, BfrB, coded by PA3531 (Yao et al., 2022). The relative proportion of FtnA and BfrB subunits in Bfr is variable and depends on the availability of environmental O_2_. The *ftnA* and *bfrB* genes are scattered in the *P. aeruginosa* genome and are regulated differently: *bfrB* transcription is stimulated by iron (Palma et al., 2003), whereas the *ftnA* gene is under control of the Anr transcription regulator, which functions to enable the adaptation of *P. aeruginosa* cells to microoxic and anoxic growth (Hammond et al., 2015). Mobilization of Fe^3+^ stored in Bfr, which is required for metabolic homeostasis in *P. aeruginosa* (Punchi Hewage et al., 2020), requires binding of a ferredoxin (Bfd), which transfers electrons to the Fe^3+^ mineral in the Bfr interior cavity for subsequent mobilization of Fe^2+^ to the cytosol (Weeratunga et al., 2009; Yao et al., 2012). Blocking the Bfr-Bfd complex in a *P. aeruginosa* Δ*bfd* mutant leads to an irreversible accumulation of iron in Bfr (Eshelman et al., 2017), and concomitant intracellular iron limitation, dysregulated carbon and sulfur metabolism, depleted amino acid biosynthesis (Punchi Hewage et al., 2020), and inability to mature and maintain biofilms (Soldano et al., 2020). The significant consequences of blocking the Bfr-Bfd complex on *P. aeruginosa* fitness motivated the discovery of small molecule inhibitors of the BfrB-Bfd complex (Punchi Hewage et al., 2019). These inhibitors have been shown to kill biofilm-embedded *P. aeruginosa* (Soldano et al., 2021), therefore suggesting the mobilization of iron stored in Bfr as a viable new target for antibiotic discovery (Rivera, 2023).

Much less is known about the function of the other two proteins thought to participate in iron storage, the products of genes PA0962, and PA4880. A recent investigation has demonstrated that the product of gene PA0962 is a Dps (DNA binding protein under starvation) (Rajapaksha et al., 2023), but nothing is known about the product of gene PA4880, which is annotated as a putative bacterioferritin. Hence, the objective of this investigation was to characterize biochemically and structurally the product of gene PA4880. We found that gene PA4880 does not encode bacterioferritin; rather, the product of gene PA4880 is a protein exhibiting hybrid structural characteristics that are typical of Dps and bacterioferritin. Proteins with these structural characteristics have been termed Dps-like, so the product of gene PA4880 in *P. aeruginosa* PAO1 is a Dps-like protein, abbreviated here as Pa DpsL.

DNA binding proteins from starved cells (Dps) are thought to be expressed in response to different stressors, including nutrient starvation, osmosis, acidic and basic environments, thermal shock, iron-induced toxicity, UV- and gamma radiation, and oxidative stress (Nair and Finkel, 2004; Calhoun and Kwon, 2011; Guerra et al., 2021). Dps are thought to protect DNA by binding and shielding, and by preventing oxidative stress caused by H_2_O_2_ in the presence of iron. Dps are 12-mer assemblies of identical subunits with a nearly spherical shell-like structure (∼9 nm diameter) containing a hollow cavity with (∼5 nm diameter) (Guerra et al., 2021; Rivera, 2023). The smaller dimensions relative to the 24-mer ferritin and bacterioferritin molecules is the reason Dps are also known as mini ferritins. The Dps subunit fold consists of a four helix bundle similar to the subunit fold of ferritin and bacterioferritin but with some unique characteristics (Rivera, 2023): (*i*) The subunits of Dps have an N- or C-terminal tail not present in ferritin or bacterioferritin, (*ii*) the long loop connecting helices B and C in the Dps four helix bundle contains a short α-helix, which is absent in ferritin and bacterioferritin, and (*iii*) Dps harbor two di-iron ferroxidase centers at the interface of each subunit dimer, where the ferroxidase center ligands are contributed by residues from both subunits. These ferroxidase centers are distinct from the ferroxidase centers in ferritin and bacterioferritin, which are buried in the middle of each subunit.

A possible different subgroup, or even possibly an outgroup of the ferritin group is the so-called Dps-like (DpsL) proteins (Lundin et al., 2012), initially characterized from the hyper thermophilic acidophile *Sulfolobus solfataricus* (Wiedenheft et al., 2005; Gauss et al., 2006) and then from the anaerobe *Bacteroides fragilis* (Gauss et al., 2012). Crystallographic studies of DpsL showed a quaternary structure (12-mer) characteristic of Dps. However, unlike Dps, where the ferroxidase centers are located at the interface of subunit dimers, the ferroxidase centers of DpsL are buried within the four-helix bundle of each subunit, akin to the ferroxidase centers in ferritin or bacterioferritin. We have cloned the PA4880 gene, overexpressed the coded protein in *E. coli* host cells and characterized the recombinant protein in solution, and by X-ray crystallography. Our findings reveal a protein with a quaternary dodecameric assembly where each subunit exhibits a four-helix bundle fold characteristic of Dps. Unlike Dps, however, the ferroxidase centers are buried within each subunit, in a manner reminiscent of bacterioferritin. Consequently, gene PA4880 encodes a DpsL protein.

## 2 Results and discussion

### 2.1 Purification and the oligomerization state of Pa DpsL in solution

**FIGURE 1:**
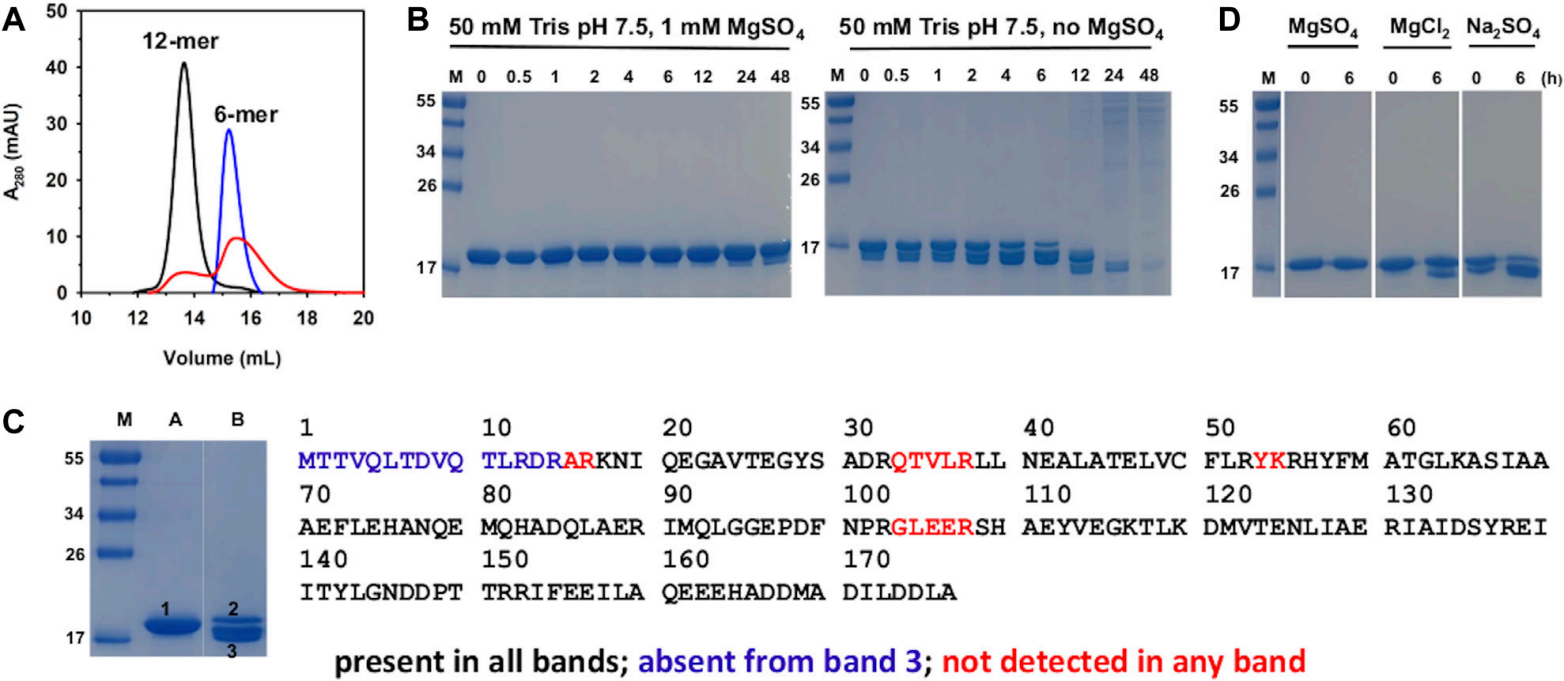
Biochemical characterization of Pa DpsL in solution. **(A)** The oligomeric state of Pa DpsL was assessed with the aid of a calibrated size exclusion column. Pa DpsL in 50 mM Tris pH 7.5 and 1 mM MgSO_4_ elutes as a 12-mer (black trace). Removal of the MgSO_4_ from the buffer in the sample and from the chromatography buffer causes the disassembly of the 12-mer (blue trace). Addition of 1 mM MgSO_4_ to the protein eluting in the blue trace chromatogram and to the chromatography buffer does not enable the full reassembly of 12-mer (red trace). **(B)** SDS PAGE shows that incubating Pa DpsL in the presence of MgSO_4_ stabilizes Pa DpsL; in the absence of MgSO_4_ the protein is proteolytically cleaved and degraded. **(C)** In gel digestion and LC-MS/MS analysis of Pa DpsL. SDS PAGE lane **(A)** 15 μM Pa DpsL in 50 mM Tris (pH 7.5) + 1 mM MgSO_4_. Lane **(B)** 15 μM Pa DpsL incubated for 6 h in 50 mM Tris (pH 7.5). Lane M: molecular weight marker. Bands 1, 2 and 3 were excised, the protein digested with trypsin, and the peptides analyzed by LC-MS/MS. All the expected peptides were detected in bands 1 and 2, indicating that the protein is intact, whereas the residues shown in blue were not detected in band 3, indicating that proteolytic cleavage starts at the amino terminus. **(D)** Incubating Pa DpsL in 50 mM Tris with MgSO_4_, MgCl_2_, or Na_2_SO_4_ for 6 h prior to SDS PAGE shows that both Mg^2+^ and SO_4_^2-^ are required to prevent proteolytic cleavage.

In the initial purification experiments, the host *E. coli* cells were lysed in 50 mM potassium phosphate buffer (pH 7.0), and the clarified lysate solution was separated on a HiTrap™ Q FF anion exchange column using the same buffer and a NaCl gradient. Fractions containing Pa DpsL were then chromatographed on a Superdex™ 200 Increase 10/300 GL size exclusion column equilibrated and eluted with 50 mM potassium phosphate (pH 6.2). Although these conditions allowed isolation of pure protein, the yield was low, and the protein was isolated as a mixture of 12-mer (small minority) and several other lower oligomerization states. Moreover, analysis of the fractions with the aid of SDS PAGE showed that the protein eluting as lower oligomerization states appeared to be cleaved. As will be discussed in Section 2.2, the fractions containing 12-mer were used in initial crystallization conditions that led to the structure of a dimer. Subsequent screening for conditions to enable purification of the protein as a 12-mer in high yield led us to observe that the presence of ≥1 mM MgSO_4_ in the buffer is required to stabilize the 12-mer assembly and to prevent cleavage of the subunits. Consequently, in subsequent work the buffers used for cell lysis, chromatography and protein storage were supplemented with at least 1 mM MgSO_4_. Dodecameric Pa DpsL was purified at pH 7.5 in three chromatographic steps, initiated by the fractionation of cell lysate on a HiTrap™ QFF anion exchange column, followed by the separation of fractions containing Pa DpsL on a second anion exchange column (Source™ 15Q) and then through a calibrated Superdex™ 200 Increase 10/300 GL size exclusion column. Fractions containing pure Pa Dps (assessed by SDS PAGE) exhibit an elution volume (Ve) corresponding to dodecameric Pa DpsL (Figure 1A, black trace).

The effect of MgSO_4_ on the dodecameric assembly of Pa DpsL was investigated by exchanging the protein into 50 mM Tris (pH 7.5), followed by sieving the resultant solution through a calibrated Superdex^TM^ 200 column. The resulting chromatogram (Figure 1A, blue trace) shows that in the absence of MgSO_4_ the 12-mer PaDpsL disassembles into lower oligomeric states. To determine whether the assembly/disassembly process is reversible, the fractions containing disassembled Pa DpsL were pooled, reconstituted with 1 mM MgSO_4_, equilibrated, and chromatographed through the same Superdex size exclusion column equilibrated with 50 mM Tris (pH 7.5) containing 1 mM MgSO_4_. The chromatogram (Figure 1A, red trace) shows only partial reassembly of the 12-mer. This observation is different from that made with 12-mer Pa Dps, which disassembles in the absence of MgSO_4_, but can be fully reassembled into 12-mer when MgSO_4_ is added (Rajapaksha et al., 2023). To investigate the nature of the irreversible disassembly of Pa DpsL we resorted to SDS PAGE and mass spectrometry. To this end, solutions of Pa DpsL in 50 mM Tris pH 7.5 in the presence and in the absence of 1 mM MgSO_4_ were incubated at room temperature for different times prior to freezing and subsequent analysis by SDS PAGE. The results (Figure 1B) show that in the absence of MgSO_4_ the Pa DpsL subunits undergo proteolysis, so it is highly probable that disassembly of the 12-mer quaternary structure is related to subunit proteolytic cleavage. In contrast, when MgSO_4_ is present, proteolytic cleavage of the subunits is largely eliminated. Hence, the stability imparted by MgSO_4_ on the integrity and fold of the subunits is probably the reason the 12-mer quaternary structure is retained when MgSO_4_ is present.

Having established that MgSO_4_ is required to prevent proteolysis of the subunits, we set out to investigate the site of proteolytic cleavage. For these experiments we excised each of the bands labeled 1-3 from the SDS PAGE gel in Figure 1C, which corresponds to a 6 h incubation time, in the presence (lane A) and in the absence (lane B) of MgSO_4_. The samples were subjected to in-gel digestion with trypsin, and the resultant peptides analyzed by LC-MS/MS. Results from these experiments carried out with band 1 (lane A) showed that all the peptides expected from trypsin digestion are detected, therefore corroborating that the Pa DpsL subunits remain intact when MgSO_4_ is present in the buffer. A similar observation was made with the protein in band 2 (lane B), which corresponds to the relatively small portion of DpsL that has not been cleaved after 6 h of incubation. In contrast, analysis of the peptides in band 3 (lane B) highlights the absence of the N-terminal peptide (blue in Figure 1C), indicating proteolytic cleavage at the N-terminal domain. It was still unclear if Mg^2+^, SO_4_^2-^, or both ions are required to prevent proteolytic cleavage and stabilize the 12-mer quaternary structure. This issue was explored by supplementing the 50 mM Tris buffer with 1 mM MgCl_2_ or 1 mM Na_2_SO_4_ and assessing the integrity of the subunits with the aid of SDS PAGE after 6 hours of incubation. The results (Figure 1D) revealed that both Mg^2+^ and SO_4_^2-^ are required for preventing cleavage of the DpsL subunit. It is also interesting to note that proteolytic degradation appears to be faster in the presence of only SO_4_^2-^ than in the presence of only Mg^2+^. The possible roles played by Mg^2+^ and SO_4_^2-^ ions in stabilizing the integrity of the Pa DpsL subunits and the quaternary structure will be discussed in Section 2.2, where the crystal structure of Pa PpsL is presented.

### 2.2 Crystal structure of a Pa DpsL subunit (Pa DpsL dimer)

**FIGURE 2:**
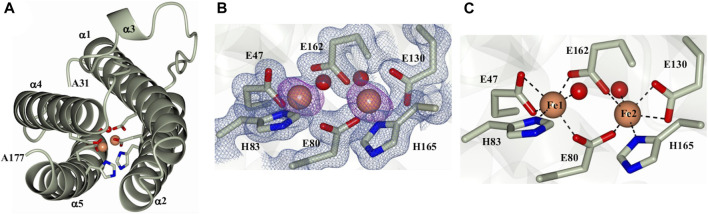
Structure of a Pa DpsL subunit (Pa DpsL-dimer). **(A)** Ribbons rendering of the model illustrating the secondary structure elements along with water molecules (red spheres), iron ions in the ferroxidase center (orange spheres), and iron-coordinating residues (green cylinders). **(B)** 2Fo-Fc electron density map (blue mesh) contoured at 1σ showing the ferroxidase center ligands and phased anomalous difference map (purple mesh) contoured at 3σ for the Fe ions (orange spheres). **(C)** Coordination of the Fe ions in the ferroxidase center.

As indicated above, the initial protein purification strategy, which was carried out in the absence of MgSO_4_, resulted in a very low protein yield containing only a small proportion of dodecamer Pa DpsL. Fractions containing 12-mer were concentrated in 50 mM phosphate buffer pH 6.2 for crystallization screens. Prismatic crystals were obtained from the Proplex screen condition D5 (8% (w/v) PEG 6,000, 100 mM MES pH 6.0, 100 mM MgCl_2_). Structure solution and refinement (Supplementary Table S1) produced a DpsL model (1.3 Å resolution) containing one molecule in the asymmetric unit, with electron density defining residues Ala31 to Ala171. The N-terminal residues Met1 to Ser30 could not be modeled due to conformational disorder. The Pa DpsL structure adopts a 4-helix bundle (Figure 2A) comprised of α1 (R33-G63), α2 (S67-Q93), α4 (L119-L144), and α5 (R152-L176), and the short helix α3 (L105-R108) in the middle of the long loop connecting α2 and α4. These structural characteristics are typical of the Dps fold. The anomalous scattering difference map revealed the presence of two large electron density peaks (36.7 σ and 34.6 σ), which were assigned as Fe ions based on the electron density maps and coordination distances. Moreover, refinement with Zn instead of Fe in the model produced negative electron density peaks at these sites. The Fe ions form a dinuclear center buried in the middle of the 4-helix bundle, where they are coordinated by the side chains of residues E47, E80, H83, E130, E162, H165, and two water molecules (Figures 2B,C). The structure of the dinuclear center, which is contained within the four-helix bundle, is different from the typical architecture of dinuclear iron centers in Dps, but nearly identical to the ferroxidase center of *P. aeruginosa* bacterioferritin (BfrB) (Weeratunga et al., 2010). Taken together, the structural features of Pa DpsL, which revealed a classic Dps fold harboring a bacterioferritin-type ferroxidase center, indicate that the product of gene PA4880 is a Dps-like protein (Pa DpsL). In agreement, superposition of the Pa DpsL structure dimer generated by the crystallographic symmetry operation–x+2, y, -z, as determined using PISA (Ciccone et al., 2015) with a dimer from the dodecameric DpsL from *B. fragilis* (PDB 2VZB) results in RMSD = 1.41 Å between C_α_ atoms (284 residues).

### 2.3 Crystal structure of a Pa DpsL dodecamer (Pa DpsL-dd and Pa DpsL-dd-Mg)

**FIGURE 3:**
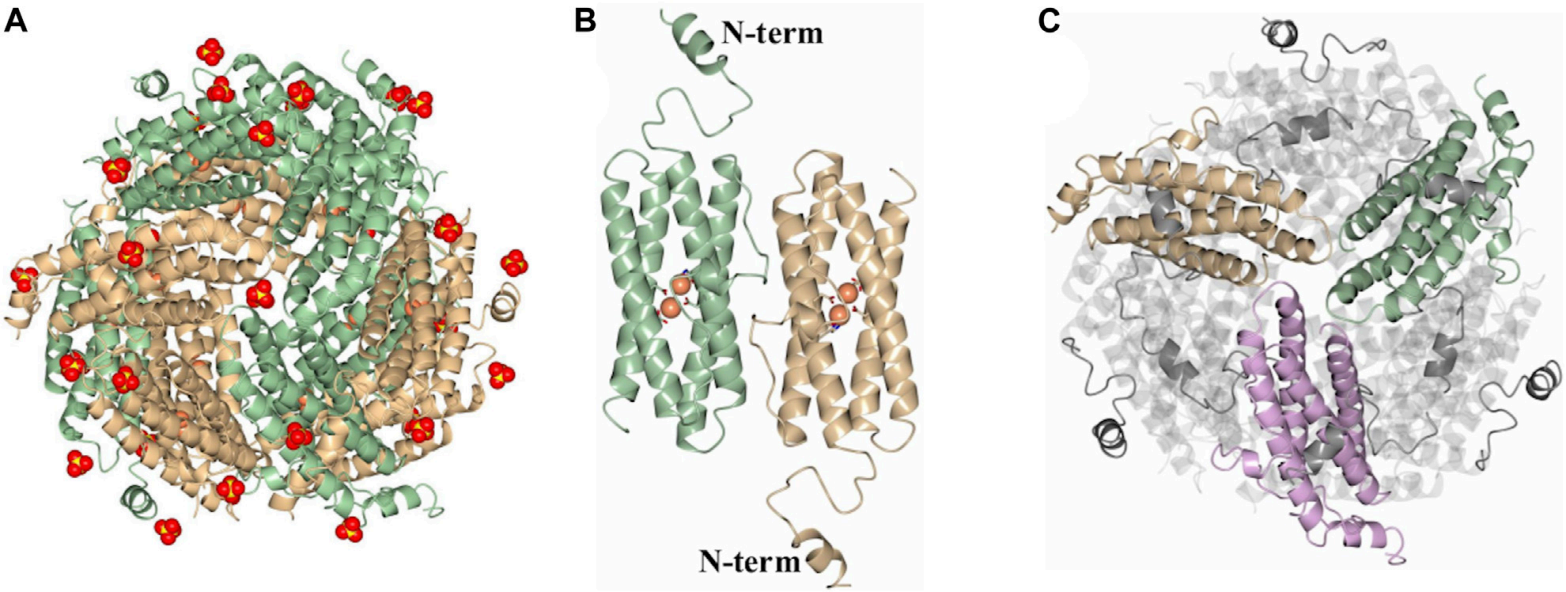
Structure of the SO_4_^2-^-bound dodecamer PaDpsL (Pa DpsL-dd). **(A)** View of the 12-mer along a 3-fold pore. The Fe and SO_4_^2-^ ions are rendered as orange and red/yellow spheres, respectively. **(B)** View of a subunit dimer in the 12-mer showing the N-terminal tails. The Fe ions and coordinating ligands in the ferroxidase centers are rendered as orange spheres and sticks, respectively. **(C)** View along an A-type 3-fold pore formed by the tan, green and violet subunits, highlighting the N-terminal tails which are positioned over neighboring subunits.

As indicated above, protein purification in the presence of MgSO_4_ enabled isolation of dodecameric DpsL in high yield. The protein was used to screen crystallization conditions leading to the formation of prismatic crystals from the Salt Rx HT screen condition F9 (800 mM lithium sulfate, 100 mM Tris pH 8.5). X-ray diffraction from these crystals, followed by structure solution and refinement produced the model termed Pa DpsL-dd (2.9 Å resolution), which contains 12 molecules (a dodecamer) in the asymmetric unit, 33 sulfate ions and 24 Fe ions (Figure 3A). The sulfate ions are in the 3-fold pores and near sites containing R13, R33, and R103. Each of the subunits exhibit a Dps fold identical to that depicted in Figure 2A, except that the position of N-terminal residues D8–S30 is made evident in the 12-mer structure by well-defined electron density (Figure 3B). This reveals that the N-terminal tails are directed toward the surface of the 12-mer where they interact with an adjacent subunit (Figure 3C). Each of the subunits harbors a di-Fe center structurally identical to the ferroxidase center *of P. aeruginosa* bacterioferritin, where the iron ions are coordinated as illustrated in Figure 2C. Consequently, the structure of the dodecamer corroborated that each of the subunits in Pa DpsL exhibit a characteristic Dps fold that harbors a ferroxidase center typical of bacterioferritin.

Prismatic crystals were also obtained from the Salt RxHT screen condition G2 (1 M MgSO_4_, 100 mM Bis-Tris pH 7.0). Structure solution and refinement produced the model termed Pa DpsL-dd-Mg (3.0 Å resolution), which contains 44 sulfate ions, 24 Fe ions, and 37 Mg ions. The locations of the Mg^2+^ ions were identified by analyzing the Fo-Fc difference electron density following refinement and by computing a Fo-Fo (Agirre et al., 2023) electron density difference map to compare the differences between the DpsL-dd-Mg and DpsL-dd data sets. Except for the presence of Mg^2+^, the structural features observed in the Pa DpsL-dd-Mg are nearly identical to those observed in the Pa DpsL-dd structure. The sites where Mg^2+^ and SO_4_^2-^ ions bind on these structures, as well as the possible significance of these bound ions to the stability of Pa DpsL is presented in section 2.4.

### 2.4 Mg^2+^ and SO_4_^2-^ in the quaternary structure of Pa DpsL

**FIGURE 4:**
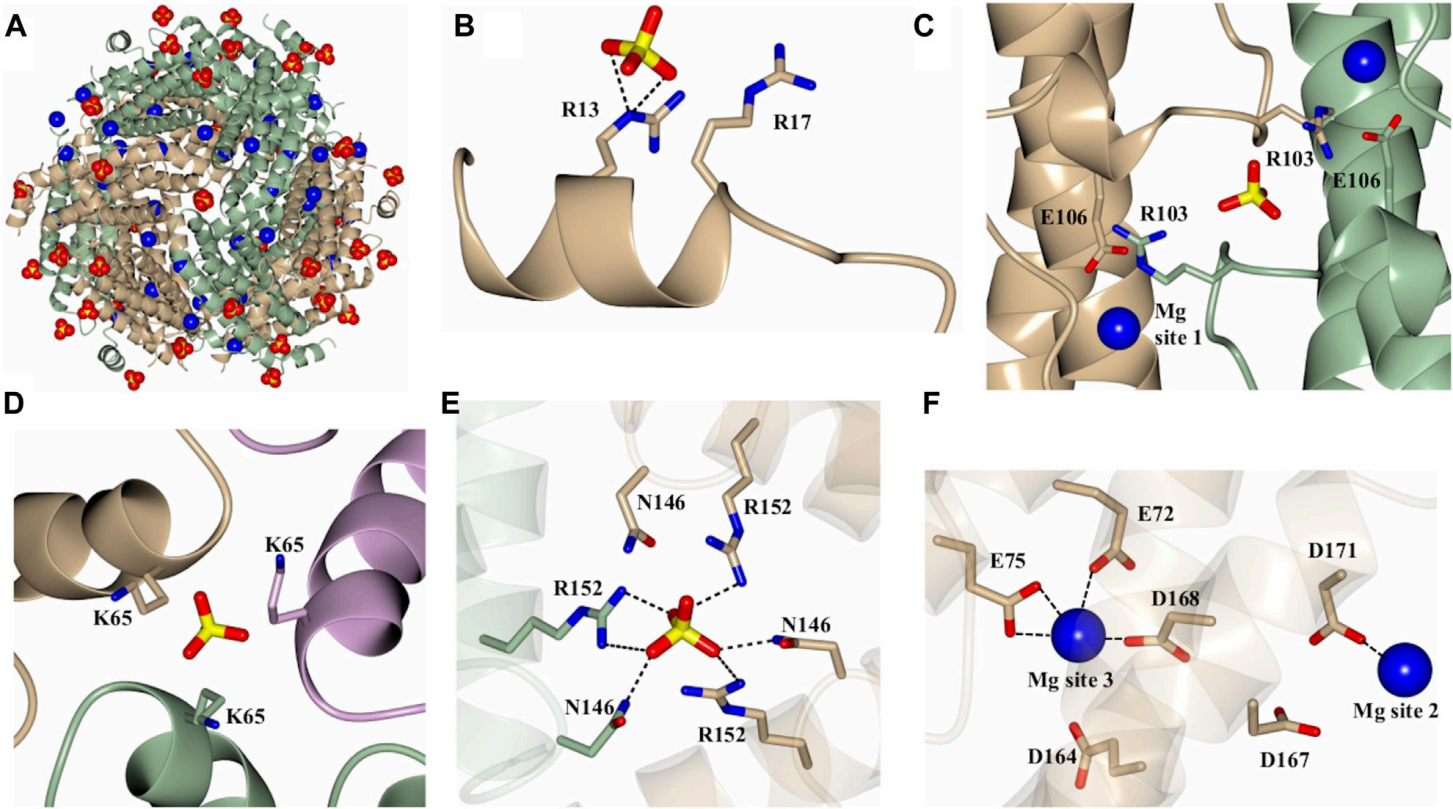
Structure of the Mg^2+^ and SO_4_^2-^ bound dodecamer Pa DpsL (Pa DpsL-dd-Mg). **(A)** View of the 12-mer showing bound Mg^2+^ (blue spheres) and SO_4_^2-^(red/yellow) ions. **(B)** Sulfate site 1 on the N-terminal tail. **(C)** Magnesium site 1 and sulfate site 2 located near the short helix (α3) on the loop connecting α2 and α4. **(D)** Sulfate site 3 bound to K65 in a type-A 3-fold pore. **(E)** Sulfate site 4 in a type-B 3-fold pore, where the sulfate ion is coordinated by the side chains of N146 and R152. **(F)** Magnesium sites 2 and 3 located approximately 13 Å from one another.

As indicated above, a network of Mg^2+^ and SO_4_^2-^ ions are observed in the structures of 12-mer Pa DpsL (Pa DpsL-dd and Pa DpsL-dd-Mg). These ions probably exert a stabilizing role of the secondary, tertiary, and quaternary structures of the protein. Four distinct SO_4_^2-^ and three unique Mg^2+^ binding sites have been observed. Both types of ions can be visualized in the Pa DpsL-dd-Mg structure (Figure 4A) The SO_4_^2-^ binding sites can be observed in sites 1–4. Sulfate site 1 is located next to each N-terminal extension, where the electrostatic interactions between SO_4_^2-^ and the side chains of Arg13 and Arg17 stabilize the two-turn helix observed in the N-terminal extension (Figure 4B). Sulfate site 2 is at the interface of two subunits where the SO_4_^2-^ ion is located between the two one-turn helices (α3) present in the long loop connecting helices α4 and α5, where the anion is electrostatically coordinated by the side chains of Arg 103 (Figure 4C). Two Mg^2+^ ions (Mg site 1) are in proximity to sulfate site 2, where each of the cations is coordinated by the side chain of Glu 106, which stems from each of the one-turn α3 helices (Figure 4C). Three-fold axes traverse the 12-mer Pa DpsL, each passing through a pair of three-fold pores formed at the intersection of three subunits. As is typical of the Dps quaternary structure, each of the two three-fold pores along a three-fold axis has a different microenvironment, thus resulting in two types of three-fold pores, termed here A and B. Each of the type-A three-fold pores, which are formed by residues near the turn separating α1 and α2 contain a SO_4_^2-^ (site 3), which interacts electrostatically with the three Lys65 residues at the pore (Figure 4D). The B-type pores (also known as ferritin-like pores), which are formed by residues near the turn separating α4 and α5, contain sulfate site 4, where the SO_4_^2-^ is coordinated to Asn146 and Arg152 (Figure 4E). Mg sites 2 and 3 are in relative proximity to one another (Figure 4F). Mg site 2 is near the protein surface where it is coordinated by the side chains of Asp 167 and Asp 171 in the C-terminal helix (α5), whereas Mg site 3 is in the 12-mer interior, where it is coordinated by the side chains of Asp164, Asp168 in the C-terminal helix and Glu 72 and Glu75 in helix α2. This extensive network of Mg^2+^ and SO_4_^2-^ ions coordinated to 12-mer DpsL suggest that the nature of the stabilizing effect that Mg^2+^ and SO_4_^2-^ have on the DpsL dodecamer structure originate from the mediation of otherwise repulsive electrostatic interactions that would take place among positively charged residues in the three-fold pores, or among the high density of negative charge concentrated near the C-terminus. Hence, in the absence of Mg^2+^ and SO_4_^2-^, one can envision significant folding-unfolding transitions affecting secondary, tertiary, and quaternary structure, which largely destabilize not only the 12-mer structure but also lead to proteolysis of the N-terminal domain. In the context of the stabilizing effect that 1 mM MgSO_4_ has on the Pa DpsL structure, it is interesting to note that the reported concentration of Mg^2+^ in bacteria is 0.5–2.0 mM (Shin et al., 2014), and that sulfate is the second most abundant soluble oxyanion after phosphate in bacterial cells (Silver and Walderhaug, 1992). Consequently, given the electrostatic role played by Mg^2+^ and SO_4_^2-^ in the stabilization of the Pa DpsL dodecamer *in vitro*, it is probable that Mg^2+^ and SO_4_^2-^ play a similar role in the *P. aeruginosa* cell, probably aided by other ions such as Ca^2+^ and phosphate.

### 2.5 Pa DpsL catalyzes the oxidation of Fe^2+^ and compartmentalizes Fe^3+^ in its core

Dps and Dps-like proteins are thought to protect cells from stressors such as oxidative pressure, nutrient limitation, and even radiation, *via* two types of activity: (*i*) the catalytic oxidation of Fe^2+^ at ferroxidase centers using H_2_O_2_ or O_2_ as electron acceptors, possibly compartmentalizing the Fe^3+^ in the protein interior, and (*ii*) shielding genomic DNA *via* binding and condensation. We investigated whether Pa DpsL exhibits similar types of activity. Our findings from experiments aimed at probing Fe^2+^ oxidation are presented in this section and the results from probing the interactions of Pa DpsL with DNA are presented in Section 2.6.

Analysis of iron associated with PaDpsL from several preparations showed 8–10 iron atoms/12-mer Pa DpsL. The analytical data, which agrees with the presence of iron in the ferroxidase centers, as determined by X-ray crystallography, also indicate that the interior cavity of the recombinant protein as isolated from *E. coli* cells is devoid of iron. The presence of a dinuclear iron center in Pa DpsL which is structurally similar to the ferroxidase centers in bacterioferritin, however, suggested that Pa DpsL may catalyze the oxidation of Fe^2+^ in solution and compartmentalize the resultant Fe^3+^ in its interior cavity. This issue was investigated by titrating solutions of the 12-mer Pa DpsL in 50 mM Tris (pH 7.5) containing 1 mM MgSO_4_ with aliquots delivering 50 Fe^2+^/12-mer to a total of 300 Fe^2+^/12-mer. These experiments were conducted in an anaerobic glove box using H_2_O_2_ as oxidant, or in air, with O_2_ as the electron acceptor, in a manner similar to that reported for Pa Dps (Rajapaksha et al., 2023).

**FIGURE 5:**
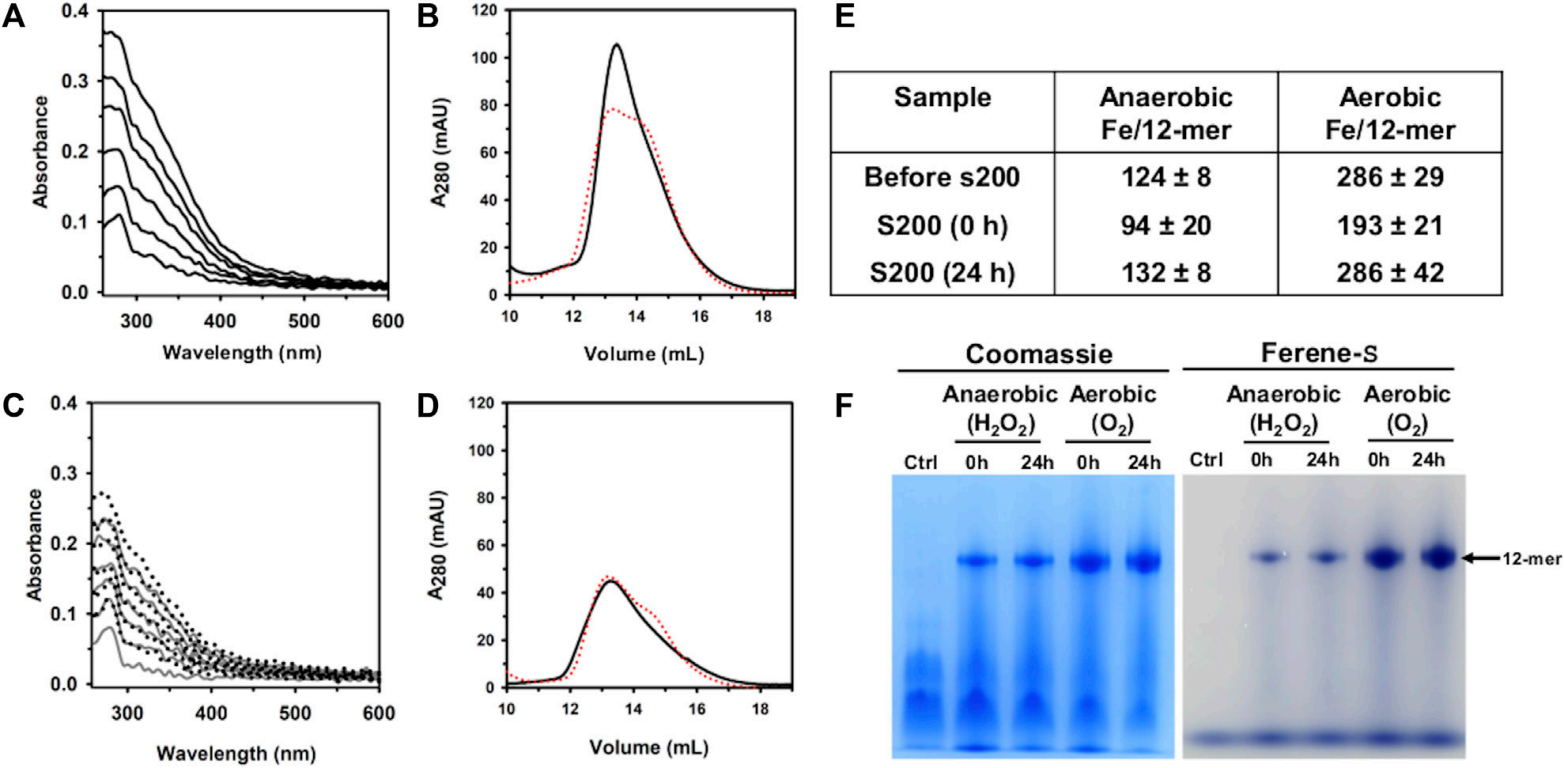
Anaerobic and aerobic mineralization of Pa DpsL. **(A)** Pa DpsL in 50 mM Tris (pH 7.5) containing 1 mM MgSO_4_ was titrated in air with aliquots of Fe^2+^ delivering 50 Fe^2+^/12-mer Pa DpsL until a total of 300 Fe^2+^ ions/12-mer had been delivered. The addition of each Fe^2+^ aliquot causes an increase in the absorbance *ca*. 320 nm due to the formation of Fe^3+^-O moieties. **(B)** Pa Dps titrated aerobically with Fe^2+^ was separated in a calibrated Superdex S200 column immediately after the titration (0 h, black trace), and after incubating 24 h at 4 °C (24 h, red trace). **(C)** Pa DpsL in 50 mM Tris (pH 7.5) + 1 mM MgSO_4_ was titrated in an anaerobic glove box, first with and aliquot of Fe^2+^ (50 Fe^2+^/12-mer) followed by addition of 1 equivalent of H_2_O_2_ relative to Fe^2+^, until 300 Fe^2+^/12-mer had been added. The addition of an Fe^2+^ aliquot does not cause changes to the spectra but the addition of an equivalent of H_2_O_2_ produces an increase in the absorbance near 320 nm. **(D)** Pa DpsL titrated anaerobically was separated in a calibrated Superdex S200 column 0 h (black) and 24 h (red) after the titration. **(E)** Iron associated with 12-mer Pa DpsL before and after passage through the Superdex S200 column. Standard deviation is from two different experiments. **(F)** Dodecamer Pa DpsL eluting from the S200 column was loaded onto a native PAGE gel, electrophorized and stained first with Ferene S to visualize iron and then with Coomassie to visualize the protein.

UV-vis spectra obtained during the titration in air (Figure 5A) show an increase in the absorbance near 320 nm following the addition of each aliquot delivering Fe^2+^, indicating the formation of Fe^3+^-O moieties. Following the addition of the last Fe^2+^ aliquot, the solution was passed through a desalting column, concentrated, and then split in two equivolumes. A small volume from each sample was used to analyze the protein and iron concentrations after the titration, and the remainder was passed through a Superdex S200 column, immediately (0 h) and after 24 h of incubation at 4 °C. The resulting chromatogram (Figure 5B) indicates that Pa DpsL remains as a 12-mer during the titration, and the content of protein and iron in the sample showed that ∼193 Fe atoms/12-mer (∼64% of the total Fe delivered) are associated with the protein eluting from the S200 column (Figure 5E). Similar analysis carried out with the sample incubated 24 h prior to passage through the S200 column showed ∼286 Fe atoms/12-mer (∼95% of the total Fe delivered).

When similar experiments were conducted in an anaerobic glove box using H_2_O_2_ as the oxidant, the UV-vis spectra acquired during the titration show no change following the addition of each Fe^2+^ aliquot (Figure 5C). Addition of an equivalent of H_2_O_2_, however, causes the absorbance near 320 nm to increase. After oxidation of the Fe^2+^ delivered with the last aliquot, the solution was desalted, concentrated, and then split in two for processing as described above for the titration in air. These experiments showed ∼94 Fe atoms/12-mer (30% of the total iron delivered) associated with the protein loaded onto the S200 column immediately after the titration, and 131 Fe atoms/12-mer (44% of the total iron delivered) when the sample was incubated 24 h prior to passage through the S200 column.

The results from the experiments carrying out the titration in air, or anaerobically using H_2_O_2_ as oxidant show that the iron content in Pa DpsL chromatographed 24 h after the titration is significantly higher than the iron content in Pa DpsL chromatographed immediately after mineralization (0 h). These observations suggest that the oxidation of Fe^2+^ at ferroxidase centers of Pa DpsL is efficient, although the process of mineralization in the interior cavity appears to be slower because non-mineralized Fe^3+^ in the 0 h sample can exit the Pa DpsL cavity and be separated from the protein in the size exclusion column. In contrast, samples passed through the size exclusion column 24 h after mineralization do not lose iron in the column, indicating that a core Fe^3+^ mineral has formed in the interior cavity.

To further probe the idea that Fe^3+^ is compartmentalized in the interior cavity of Pa DpsL, fractions eluting from the Superdex column were analyzed in a native PAGE gel, staining with Ferene S to visualize iron and then with Coomassie to visualize the protein. The results from these experiments (Figure 5F), which show that iron is indeed compartmentalized in Pa DpsL, indicate that Pa DpsL probably functions in the detoxification of iron-induced oxidative stress. In this context, although the oxidation of Fe^2+^ by Pa DpsL can be carried out with O_2_ or with H_2_O_2_ as electron acceptors, the process *in vitro* appears to be more efficient when O_2_ is the oxidant (Figure 5E). Additional studies are needed to gain detailed understanding of the Fe^2+^ oxidation catalyzed by DpsL, as well as the mineralization process leading to the formation of a compartmentalized Fe^3+^ mineral.

### 2.6 Pa DpsL cleaves DNA *in vitro*

**FIGURE 6:**
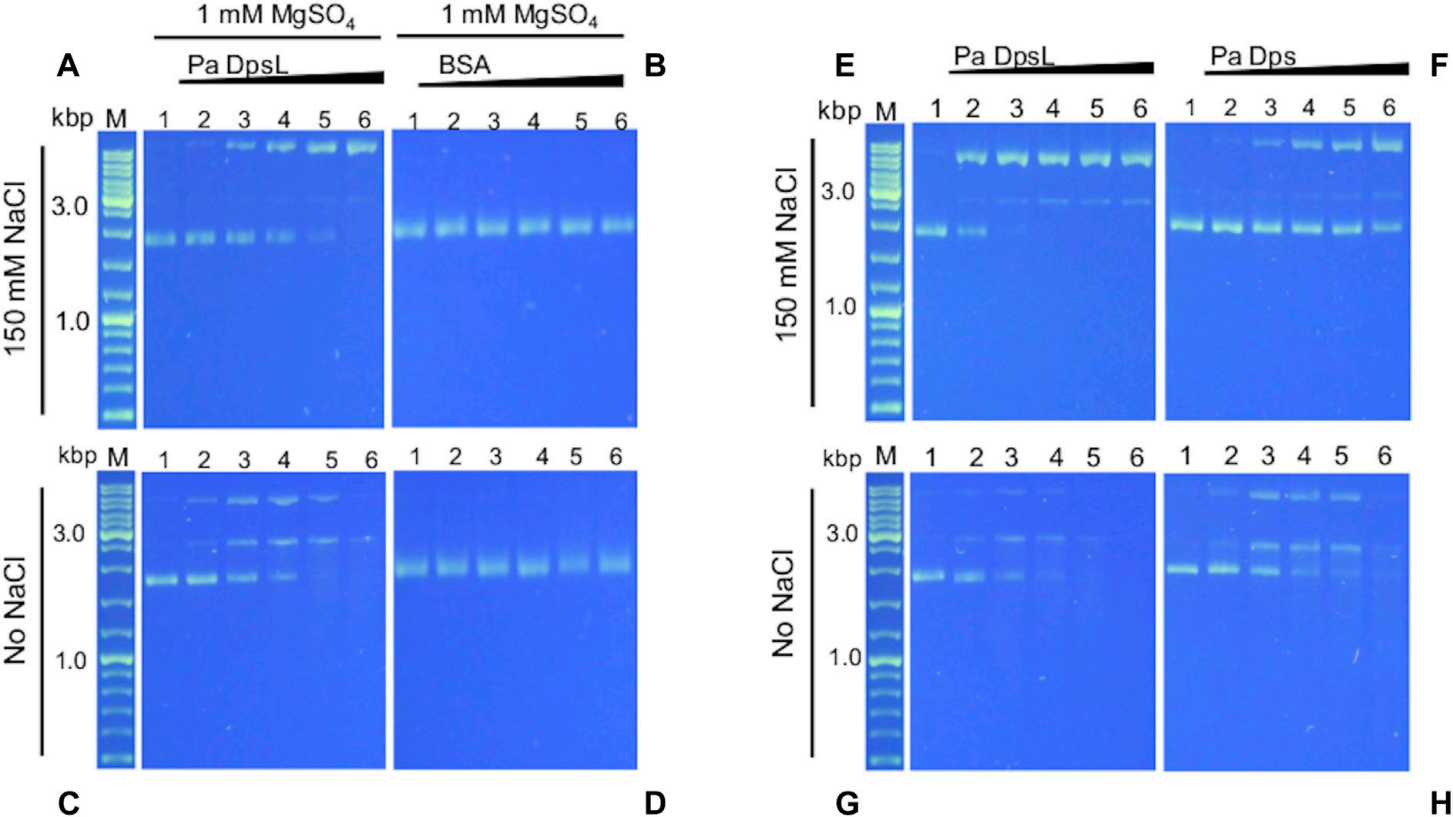
Pa DpsL cleaves plasmid DNA. pUC18 plasmid DNA was incubated (15 min, 35 °C) with different concentrations of Pa Dps in 50 mM Tris (pH 7.5) + 1 mM MgSO_4_ with or without 150 mM NaCl, and then separated in a 1% agarose gel. **(A, C)** Pa DpsL, **(B, D)** bovine serum albumin (BSA) control. Lane M = DNA ladder, lane 1 = DNA control, lanes 2-6, respectively, Pa DpsL/DNA mole ratio = 5, 25, 50, 100, 200. **(E, F)** The DNA cleaving activity of Pa DpsL and Pa Dps was compared by incubating (30 min, 35 °C) each of the proteins with plasmid DNA in 50 mM Tris (pH 7.5) + 1 mM MgSO_4_ with **(E, F)** and without **(G, H)** 150 mM NaC.l.

Dps is thought to bind DNA to protect it from the toxic effects of oxidative stress. In this context, it has been reported that *E. coli* Dps and plasmid DNA in solution form a complex too large to enter the pores of 1% agarose gels (Almiron et al., 1992). DNA binding, however, is not a ubiquitous property of Dps, since several Dps have been reported to not bind DNA (Guerra et al., 2021). In addition, DNA recognition by Dps is probably non-specific but thought to be mediated by electrostatic interactions between positively charged residues in the N- or C-terminal tails of 12-mer Dps (or DpsL) and DNA (Lee et al., 2015). To test this idea, a constant concentration of pUC18 plasmid DNA (∼2.7 kbp) was incubated for 15 min with increasing concentrations of as isolated Pa DpsL in 50 mM Tris buffer (pH 7.5) containing 150 mM NaCl and 1 mM MgSO_4_ and the resultant mixture loaded onto an agarose gel for electrophoresis. Surprisingly, the results from these experiments show that Pa DpsL nicks the plasmid DNA (Figure 6A), while in contrast, in the presence of bovine serum albumin (BSA) as control, the plasmid DNA remains supercoiled (Figure 6B).

Assuming that the interactions between Pa DpsL and DNA which result in nicking of the plasmid DNA are electrostatic in nature, the experiments were repeated at low ionic strength, by incubating the protein and pUC18 plasmid DNA in 50 mM Tris buffer, pH 7.5 containing 1 mM MgSO_4_. In these conditions, the plasmid incubated with Pa DpsL is first nicked, then linearized and ultimately degraded (Figure 6C), whereas plasmid DNA incubated with BSA remains supercoiled (Figure 6D). Similar experiments carried out by incubating Pa Dps and pET11a plasmid (∼5.7 kbp) in 50 mM Tris buffer (pH 7.5) containing 1 mM MgSO_4_ produced analogous results to those observed with the pUC18 plasmid. In the presence of 150 mM NaCl the circular plasmid was nicked, whereas at low ionic strength the plasmid was degraded (Supplementary Figure S1).

The higher DNA cleaving activity observed at low ionic strength is consistent with ionic steering of the DNA Pa DpsL interactions. In this context, as discussed in section 2.4 (Figure 4), a network of SO_4_^2-^ ions bind the dodecamer *via* electrostatic interactions. Electrostatic rendering of the dodecamer surface viewed along each of the two types of 3-fold pore (Supplementary Figure S2) suggest possible sites where the negatively charged backbone of DNA may interact with Pa DpsL, thus facilitating the nicking and cleaving activity of Pa DpsL. Additional studies are required to elucidate the mechanism of DNA nicking and degradation.

A similar plasmid DNA nicking and cleaving activity was recently reported for Pa Dps (Rajapaksha et al., 2023). Consequently, we decided to compare the activity of both proteins under identical conditions. To this end, we incubated plasmid DNA (constant concentration) with increasing concentrations of Pa Dps, or Pa DpsL in Tris buffer (pH 7.5) containing 1 mM MgSO_4_ for 30 min prior to separating the mix on agarose gels. When the reaction is carried out at high ionic strength, the plasmid DNA is completely nicked when the Pa DpsL/DNA mole ratio is 25 (Figure 6E), while in contrast, the supercoiled plasmid DNA is only partially nicked in the presence of Pa Dps, even at the higher Dps/DNA mole ratios (Figure 6F). The higher DNA cleaving activity of Pa DpsL relative to Pa Dps is also observed in the gels loaded with the reactions carried at low ionic strength, where it is evident that Pa DpsL (Figure 6G) degrades plasmid DNA more efficiently than Pa Dps (Figure 6H).

## 3 Discussion

The genome of *P. aeruginosa* encodes four types of iron storage protein, two are ferritin-like molecules (FtnA, BfrB), and two are miniferritins (Dps and DpsL). The presence of two genes coding for ferritin like molecules has been recently explained by the discovery showing that FtnA and BfrB co-assemble into a mixed subunit bacterioferritin (Bfr), with the relative proportion of FtnA and BfrB subunits dictated by environmental O_2_ availability (Yao et al., 2022). Transcription of the *bfrB* gene is induced in response to high environmental iron (Ochsner et al., 2002; Wilderman et al., 2004), while the *ftnA* gene, which is part of the Anr regulon, is transcribed in response to low environmental O_2_ (Hammond et al., 2015). Given that both the FtnA and BfrB subunits of Bfr harbor active but structurally distinct ferroxidase centers, it has been speculated that the ferroxidase center of BfrB is tuned to react rapidly with H_2_O_2_, whereas the ferroxidase center of FtnA may have a higher affinity for O_2_, which allows Fe^2+^ oxidation and Fe^3+^ storage under microaerophilic conditions (Yao et al., 2022).

The *P. aeruginosa* genome also harbors two genes encoding distinct miniferritins, Pa Dps (PA0962) and Pa DpsL (PA4880). Although the subunits of both types of protein adopt the classic Dps fold and have an N-terminal tail, they differ significantly in the structure of their ferroxidase centers. In Pa Dps (Rajapaksha et al., 2023), like in classic Dps proteins, the dinuclear iron sites of the ferroxidase centers are located at the interface of 2-fold symmetry-related subunit dimers, and the iron ligands, which are highly conserved amongst Dps sequences, are contributed by each of the subunits in the dimer (Ilari et al., 2000; Ilari et al., 2002; Rajapaksha et al., 2023). In Pa DpsL the ferroxidase center dinuclear site and corresponding ligands are contained within each of the subunits. Interestingly, the ferroxidase center ligands in Pa DpsL are identical to those of Pa BfrB, which is probably one reason the PA4880 gene in the *P. aeruginosa* PAO1 genome has been annotated as coding a “possible bacterioferritin”. The structural characterization of the product of gene PA4880 presented here shows a protein with a Dps fold but with bacterioferritin-like ferroxidase centers. Hence, we propose that gene PA4880 should be annotated as encoding a Dps-like protein (*dpsL*).

Although it is now clear that the *P. aeruginosa* genome encodes two types of Dps proteins, it is not yet completely understood why the two types of Dps are needed. Pa Dps has been implicated in the defense against oxidative stress and in a protective role against the β-lactam antibiotic Meropenem, possibly due to its reactive oxygen species (ROS) suppressing activity (Maura et al., 2016). Pa Dps has also been implicated in a H3-T6SS-mediated protection of *P. aeruginosa* cells against H_2_O_2_ by its predicted ability to catalyze the oxidation of Fe^2+^ and compartmentalize Fe^3+^ in its interior cavity (Lin et al., 2023). These conclusions are supported by the more recent structural and biochemical characterization of Pa Dps, which demonstrated catalytic ferroxidase activity, competency to store Fe^3+^ in its interior cavity, and the ability to protect *P. aeruginosa* cells from H_2_O_2_-induced toxicity (Rajapaksha et al., 2023). Even less is known about Pa DpsL. The *dpsL* gene (PA4880) is expressed under iron-replete conditions and is regulated by the small regulatory RNAs PrrF1 and PrrF2 (Wilderman et al., 2004). In agreement, proteomic analysis of the *P. aeruginosa* iron starvation response imposed by limiting iron in the media (Nelson et al., 2019), or by irreversibly trapping iron in bacterioferritin (Punchi Hewage et al., 2020), results in low abundance of Pa DpsL. The transcriptional response of *dpsL* to environmental iron levels is also in agreement with the structure of Pa DpsL, which harbors ferroxidase centers to catalyze the oxidation of Fe^2+^ and an interior cavity that can compartmentalize Fe^3+^. Taken together, the transcriptomic, proteomic, and structural studies converge on a role for Pa DpsL in the protection toward iron-induced toxicity. Pa DpsL, however, may play additional roles in the cell, since transcriptomic analyses have also implicated the *dpsL* gene in the osmotic stress (Aspedon et al., 2006), and in the quorum sensing (Schuster et al., 2003) response of *P. aeruginosa*.

It is also of note that Pa DpsL, like Pa Dps (Rajapaksha et al., 2023), can efficiently nick DNA. In this context, restriction-modification systems, consisting of a restriction endonuclease and cognate methyl transferase, are considered innate immune mechanisms that protect their host against foreign DNA (Santoshi et al., 2023). Nicking endonucleases, like restriction endonucleases, recognize a short specific sequence in double-stranded DNA and cleave it at a specific position relative to the recognition sequence. Unlike restriction endonucleases, however, nicking endonucleases cleave only one DNA strand (Zheleznaya et al., 2009). Consequently, it is possible that Pa DpsL, in addition to protecting the *P. aeruginosa* against iron-induced toxicity, may contribute to protecting the cell against invading foreign DNA. Although the work presented here demonstrates that Pa DpsL can efficiently nick plasmid DNA, and at higher concentrations restrict and degrade DNA, it is not yet known whether a specific sequence is recognized. It is also interesting that both miniferritins in *P. aeruginosa*, Pa Dps (Rajapaksha et al., 2023), and Pa DpsL exhibit nuclease activity. This suggests that miniferritins from other bacteria may also restrict DNA. Future work aimed at probing whether Pa DpsL and Pa Dps recognize specific DNA sequences, as well as searching for other miniferritins exhibiting nuclease activity will help understand how the nuclease activity of these molecules contributes to the bacterial immune response and fitness.

## 4 Materials and methods

### 4.1 Chemicals

Unless otherwise specified chemicals were purchased from Fisher Scientific (Waltman, MA, United States of America). The gene encoding Pa DpsL (PA4880) was synthesized, subcloned into a pET11a vector, and sequenced (GeneScript Corp., Piscataway, NJ). The gene was engineered with silent mutations introducing codons favored by *E. coli* (Ikemura, 1985), and with NdeI and BamHI restriction sites at the 5′ and 3’ ends, respectively. The pET11a-dpsL construct was transformed into *E. coli* BL21-Gold (DE3) cells (Agilent Technologies) for protein expression.

### 4.2 Expression and purification of Pa DpsL

Pre-cultures were grown overnight from a single colony of *E. coli* BL21-Gold (DE3) cells transformed with the pET11a-*dpsL* construct in LB media containing 50 μg/mL ampicillin. The pre-cultures were used to inoculate 1L of fresh LB media, and the expression cultures were induced with 0.5 mM isopropyl β-D-1thiogalactopyranoside (IPTG) when the optical density at 600 (OD_600_) reached 0.6–0.8. Four hours post-induction the cells were harvested by centrifugation and stored at −80 °C until lysis. The initial purification attempts were carried out by resuspending the cell pellet in 50 mM potassium phosphate (pH 7.0) containing 0.5 mM phenylmethyl sulfonyl fluoride (PMSF), and a protease inhibitor cocktail tablet (Thermo Scientific). The cells were lysed by ultrasound in an ice bath with the aid of a Qsonica Q500 sonicator operating with a 60% pulse amplitude and 10 cycles of pulse-on (10 s) pulse-off (45 s). The cell lysate was clarified by centrifugation (63,000 x *g*, 4 °C, 45 min), loaded onto a 5 mL HiTrap™ Q FF (GE Healthcare) column equilibrated with lysis buffer, and then eluted with a 0–600 mM NaCl gradient. Fractions containing Pa DpsL were combined and loaded onto a Superdex™ 200 Increase 10/300 GL size exclusion column (25 mL, GE Healthcare) equilibrated with 50 mM potassium phosphate (pH 6.2) containing 0.5 mM PMSF, and a protease inhibitor tablet (Thermo Scientific). This protocol allowed isolation of small amounts of pure Pa DpsL which consisted of 12-mer (small minority) and lower oligomerization states. This protein was used to grow the prismatic crystals used to determine the structure of a Pa DpsL subunit (Pa DpsL-dimer).

Screening for experimental conditions led us to observe that the presence of ≥1 mM MgSO_4_ in the buffer stabilizes the 12-mer. Consequently, purification of 12-mer Pa DpsL was carried out as follows: The cell pellet was resuspended in lysis buffer: 50 mM Tris (pH 7.5), 100 mM NaCl, 0.5% Triton X-100, 2 mg/mL lysozyme, 0.5 mM (PMSF), 1 mM MgSO_4_, 1 mM CaCl_2_, and a protease inhibitor cocktail tablet. The cells were lysed with ultrasound and the lysate was clarified by centrifugation as described above. The clarified lysate was dialyzed against 50 mM Tris (pH 7.5), 3 mM MgSO_4_, 0.5 mM PMSF, and protease inhibitor cocktail tablet at 4 °C. The resultant solution was filtered through a 4 μm filter (VWR), and then loaded onto a 10 mL HiTrap™ Q FF anion exchange column, washed with the same buffer, and then eluted with a NaCl gradient (0–300 mM). Fractions containing Pa DpsL were pooled, dialyzed against 50 mM Tris (pH 7.5), 3 mM MgSO_4_, 0.5 mM PMSF, loaded onto Source™ 15Q anion exchange column (Cytiva), washed with the same buffer, and eluted with an NaCl gradient (80–280 mM). Fractions containing Pa DpsL were pooled, concentrated, and then chromatographed through a calibrated Superdex™ 200 Increase 10/300 GL size exclusion column (25 mL) equilibrated and eluted with 50 mM Tris (pH 7.5), 1 mM MgSO_4_. The protein concentration was determined with the aid of a bicinchoninic acid (BCA) assay kit (Pierce™ BCA Protein Assay Kit, Thermo Scientific) following the manufacturer provided instructions.

### 4.3 Crystallization and structure solution

All crystallization experiments were carried out using an NT8 drop setting robot (Formulatrix Inc.) and UVXPO MRC (Molecular Dimensions) sitting drop vapor diffusion plates at 18°C. 100 nL of protein and 100 nL crystallization solution were dispensed and equilibrated against 50 μL of the latter. The purified Pa DpsL-dimer was concentrated to 8 mg/mL (0.4 mM) in 50 mM phosphate buffer, pH 6.2 for crystallization screening. Prismatic crystals formed in approximately 1 week from the Proplex screen (Molecular Dimensions) condition D5 (8% (w/v) PEG 6,000, 100 mM MES pH 6.0, 100 mM MgCl_2_). The protein sample used to obtain the structure of dodecameric Pa DpsL (Pa DpsL-dd) and the dodecameric form with Mg^2+^ (Pa DpsL-dd-Mg) was concentrated to 13.3 mg/mL (0.4 mM) in 50 mM potassium phosphate pH 6.2, 0.5 mM PMSF. Prismatic crystals formed in approximately 1 week from the Salt Rx HT screen (Hampton Research) condition F9 (800 mM lithium sulfate, 100 mM Tris pH 8.5) for Pa DpsL-dd and from condition G2 (1 M MgSO_4_, 100 mM Bis-Tris pH 7.0) for Pa DpsL-dd-Mg. Samples were transferred to a cryoprotectant solution composed of 80% (v/v) crystallization solution and 20% (v/v) glycerol before storing in liquid nitrogen. X-ray diffraction data were collected at National Synchrotron Light Source II AMX beamline 17-ID-1 using an Eiger 9M pixel array detector for Pa DpsL-monomer. X-ray diffraction data for Pa DpsL-dd and Pa DpsL-dd-Mg were collected at National Synchrotron Light Source II NYX beamline 19-ID using a Dectris Pilatus 6M pixel array detector.

Intensities were integrated using XDS (Kabsch, 2010b; a) and the Laue class analysis and data were scaled together with Aimless (Evans, 2011). Data from three datasets were merged for Pa DpsL-dimer to increase the multiplicity and structure solution was conducted by SAD phasing using the ShelxCDE (Sheldrick, 2010) pipeline which yielded an estimated mean FOM of 0.657 and pseudo-free CC of 71.15% for the original enantiomorph. ShelxE autotracing produced a Cα model that contained 141 residues which was used for the automated building with Arp/warp (Langer et al., 2008). The final model, containing a monomer in the asymmetric unit was obtained by additional refinement and manual model building with Phenix (Liebschner et al., 2019) and Coot (Emsley et al., 2010), respectively. This model was used for structure solution of Pa DpsL-dd, and Pa DpsL-dd-Mg data by molecular replacement with Phaser (McCoy et al., 2007). All atoms except solvent molecules were refined with anisotropic atomic displacement parameter for Pa DpsL monomer. Disordered side chains were truncated to the point for which electron density could be observed. Structure validation was conducted with Molprobity (Chen et al., 2010) and figures were prepared suing the CCP4MG package (McNicholas et al., 2011). Structure superposition was conducted with GESMT (Krissinel, 2012). Crystallographic data are provided in Supplementary Table S1.

### 4.4 Titration of Pa Dps with Fe^2+^ under aerobic and anaerobic conditions

Anaerobic titrations were conducted in an anaerobic glove box (Coy): 12-mer Pa DpsL (1.5 mL, 2.0 μM) in 50 mM Tris (pH 7.5) containing 1 mM MgSO_4_ was placed in a container outfitted with a magnetic bar and titrated with a solution of 20 mM FeCl_2_, with each aliquot delivering 50 Fe^2+^/12-mer, followed by the addition of 1 equivalent of H_2_O_2_ relative to Fe^2+^. After the addition of each aliquot, the solution was stirred for 2 min prior to recording UV-vis spectra using a Cary50 spectrophotometer and a 0.2 cm pathlength fiber optic probe. After addition of the last aliquot (total of 300 Fe^2+^/12-mer) the solution was desalted through a Sephadex G25 M column, concentrated to 1,140 μL, and divided into two equivolume samples. A 50 μL aliquot was used to analyze the Fe content, and a 20 μL aliquot to determine the protein concentration. One of the samples (500 μL), termed 0 h, was chromatographed through a Superdex™ 200 Increase 10/300 GL size exclusion column equilibrated and eluted with 50 mM Tris (pH 7.5) containing 1 mM MgSO_4_. The second sample (500 μL) was incubated for 24 h at 4 °C and then chromatographed through the Superdex™ 200 Increase size exclusion column equilibrated and eluted with 50 mM Tris (pH 7.5) containing 1 mM MgSO_4_. Pa DpsL fractions eluting from the size exclusion column were pooled and concentrated to 500 μL, prior to sampling aliquots for the determination of Fe (50 μL) and protein (20 μL) concentrations. The remaining was concentrated to 100 μL and then used to load native and SDS PAGE gels. Aerobic titrations were carried out similarly, except that dissolved O_2_ was used as the oxidant.

### 4.5 Pa DpsL interactions with plasmid DNA

pUC18 plasmid (4 μL, 15 ng/μL) was mixed with distinct concentrations of Pa DpsL (4 μL) in 50 mM Tris (pH 7.5) containing 1 mM MgSO_4_, with or without 150 mM NaCl, to produce solutions containing Pa DpsL/pUC18 M ratio = 5, 25, 50, 100 and 200. The reactions were incubated (15 min) at 35 °C and then quenched by addition of loading dye prior to loading 1% agarose gels for electrophoresis (TBE buffer, 80 V, 105 min, room temperature). DNA bands were visualized using SYBR safe DNA staining dye.

### 4.6 Mass spectrometry

Bands 1, 2 and 3 in Figure 1C were excised from the SDS PAGE gel, and the bands processed as described previously (Yao et al., 2022). In brief, the protein in the gel bands was reduced with dithiothreitol and alkylated with iodoacetamide before overnigh incubation in buffered trypsin solution. The extracted peptides were dried, reconstituted in 0.1% formic acid and qunatified with the aid of UV-vis spectrophotometry in a Biotek Epoch2 plate reader. A total of 500 ng tryptic peptides was injected in the mass spectrometer.

Mass spectrometry was conducted on a LC-MS/MS Vanquish Neo liquid chromatography system coupled to a Q-Exactive orbitrap mass spectrometer equipped with a flex source for nano-LC (Thermo Scientific). Chromatography was conducted by first loading onto a trap cartridge (Thermo Scientific Acclaim PepMap NEO, 5 µm particle size, 1 mm i.d., 5 mm length) at 20 μL/min using an isocratic program with 98:2, H_2_O: acetonitrile (ACN) containing 0.1% formic acid. This step allowed for sample pre-concentration and desalting. Analytical separation of the trapped peptides was conducted with a nanoLC column (Thermo Scientific Acclaim PepMap, 3 µm particle size, 0.075 mm i.d., 250 mm length) coupled to a metal emitter with an i.d. of 30 µm. Separation was carried out with a gradient using two mobile phases: A = H_2_O, B = ACN, both containing 0.1% formic acid. After the initial 5 min of trapping, the gradient started at 5% B (2 min), increased to 10% B in 1 min then to 30% B in 30 min. The column was then washed with 90% B (5 min) before returning to 5% B for re-equilibration. Ionization was achieved with a constant voltage (1.9 kV) in positive ion mode, while the mass spectrometer was operated in data-dependent acquisition (DDA) mode using a loop count of 10. The chromatographic peak width was set to 45 s, the full MS resolution to 70,000 with an adjusted gain control (AGC) target of 1e^6^ and a maximum injection time (IT) of 50 ms. The scan range was set to 375–1,400 m/z. The MS/MS resolution was set to 17,500 with an AGC target of 1e^5^ and a maximum IT of 110 ms. The isolation window for the parent ion was set to 1.4 m/z while the scan range was automatically adjusted based on size and charge of the parent ion. The normalized collision energy (NCE) was set to 28. The minimum AGC to trigger an MS/MS scan was set to 1.1e^3^, which resulted in a minimum intensity threshold of 1.0e^4^. Only peaks with charges between 2 and 6 were considered for MS/MS scans. Isotopes were excluded, and all parent ions scanned were temporarily included in an exclusion list for 60 s.

Raw data were visualized with Thermo Scientific FreeStyle (ver. 1.8) to confirm peptide intensity and separation quality. Samples were then analyzed with Thermo Scientific Proteome Discoverer 2.4 using both Sequest HT and Mascot (Matrix Science, ver. 2.8) as search engines. Data were searched against the latest available Uniprot reference proteomes for *P. aeruginosa* (strain ATCC 15692/DSM 22644/CIP 104116/JCM 14847/LMG 12228/1C/PRS 101/PAO1, proteome ID UP000002438) database using a tolerance of 10 ppm for the parent ion and 0.2 m/z for the fragments. Carbamidomethylation of cysteine was selected as static modification while methionine oxidation was selected as dynamic modification. Percolator was used to calculate posterior error probabilities for each peptide spectrum match (PSM). A second database containing common contaminants was also used to filter out unrelated protein hits. A consensus workflow was employed to eliminate peptide redundancy, further validate PSM assignment and provide protein identification and FDR validation. Both proteins and peptides were assigned a high level of confidence if FDR values were lower than 0.01 and a medium level of confidence if FDR values were between 0.01 and 0.05. Finally, each protein was required to have at least 1 unique peptide.

## Data Availability

The data presented in the study are deposited in the Worldwide Protein Databank (wwPDB), with the accession codes 8W1D (Pa DpsL-monomer), 8W1E (Pa DpsL-dd) and 8W1F (Pa DpsL-dd-Mg).
