# Supplementary material for: Pseudomonas aeruginosa gene PA4880 encodes a Dps-like protein with a Dps fold, bacterioferritin-type ferroxidase centers, and endonuclease activity
Source: Front Mol Biosci. 2024 May 22;11:1390745. doi: 10.3389/fmolb.2024.1390745 (PMC11150526; doi:10.3389/fmolb.2024.1390745)
Supplement: Supplementary file 1 [file Image1.pdf]

Supplementary Material

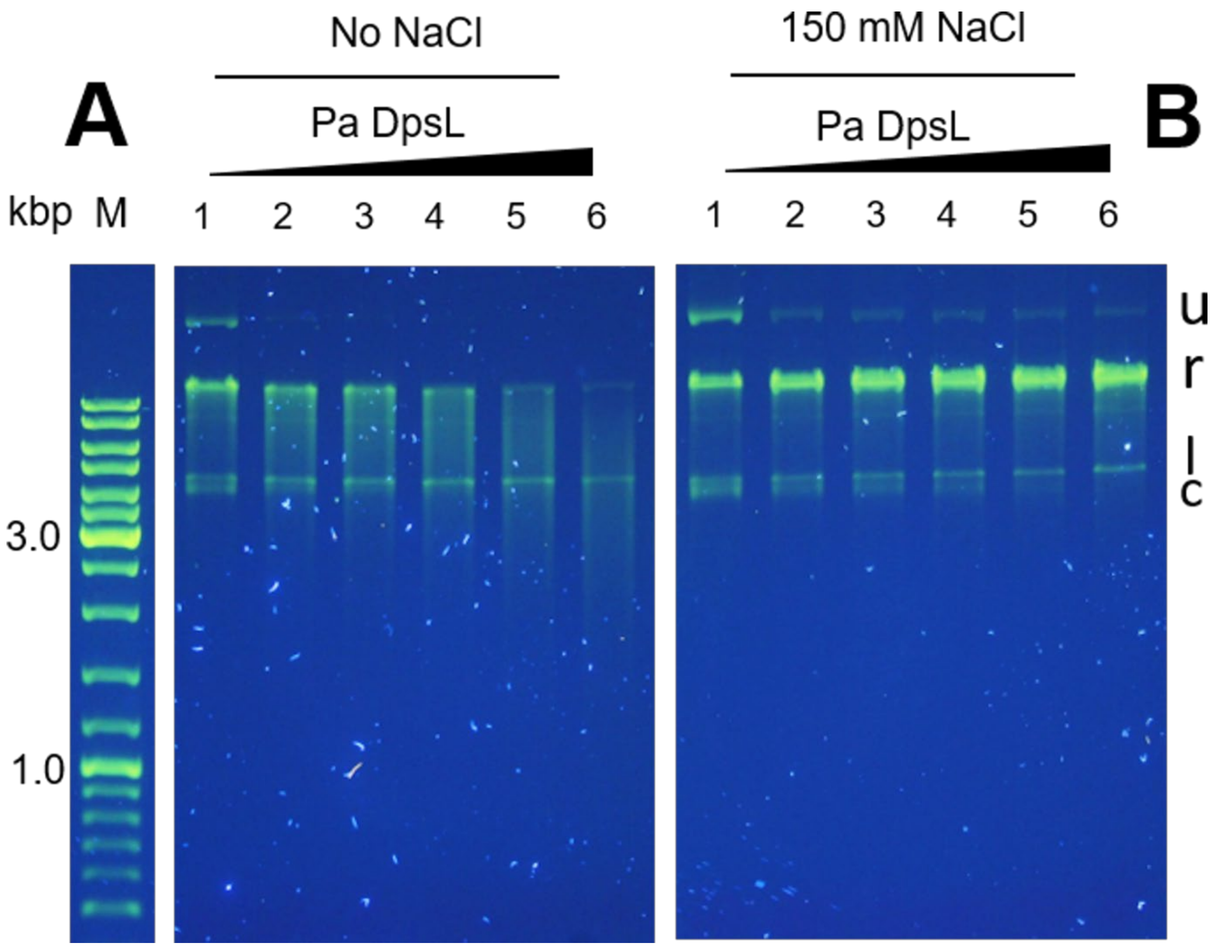

Figure S1. pET 11a plasmid DNA was incubated (60 min, 35 °C) with distinct concentrations of Pa DpsL and then separated in a 0.75% agarose gel. (**A**) Incubation in 50 mM Tris buffer (pH 7.5) containing 1 mM $MgSO_4$ causes degradation of the plasmid. (**B**) Incubation in 50 mM Tris buffer (pH 7.5) containing 150 mM NaCl and 1 mM $MgSO_4$ results mainly in nicking of the plasmid DNA. Lane 1 = DNA, lanes 2-6, respectively, Pa DpsL/DNA more ratio = 50, 100, 200, 400, 800. Lane M = DNA electrophoresis ladder. c = circular supercoiled DNA, r = relaxed or nicked DNA, l = linear DNA, u = unknown form.

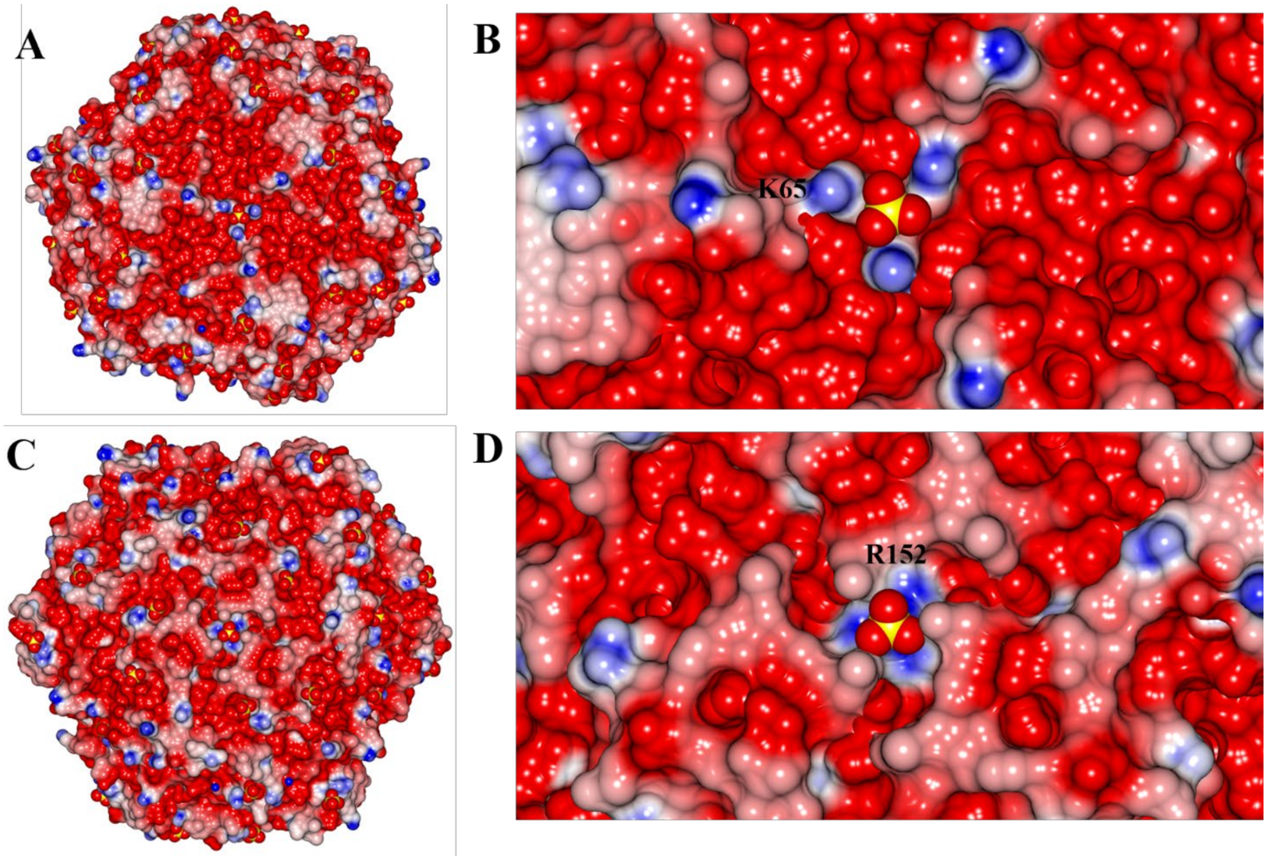

Figure S2. Electrostatic surface representation of the Pa DpsL dodecamer (-0.5 V (red) to 0.5 V (blue)). Sulfate ions are rendered as yellow/red spheres. (**A**) View along the type-A 3-fold pore and (**B**) Zoomed-in view of a type-A 3-fold pore. (**C**) View along the type-B 3-fold pore and (**D**) Zoomed-in view of a type-B 3-fold pore.

Table S1. Crystallographic Data for Pa DpsL

	Pa DpsL-monomer	Pa DpsL-dd	Pa DpsL-dd-Mg
**Data Collection**			
Unit-cell parameters (Å, °)	a=57.94, b=44.58, c=51.47, β=97.0	a=b=216.18, c=278.51	a=b=215.79, c=277.54
Space group	$C2$	$P4_32_12$	$P4_32_12$
Resolution (Å)[1]	28.76-1.30 (1.32-1.30)	49.51-2.90 (2.95-2.90)	49.94-3.00 (3.05-3.00)
Wavelength (Å)	0.9201	0.9795	0.9795
Temperature (K)	100	100	100
Observed reflections	327,050	2,082,925	1,874,799
Unique reflections	32,140	145,618	130,805
$\langle I/\sigma(I)\rangle$[1]	12.3 (1.5)	12.8 (1.7)	12.1 (1.8)
Completeness (%)[1]	100 (99.8)	100 (100)	100 (100)
Multiplicity[1]	10.2 (8.8)	14.3 (15.1)	14.3 (15.1)
R_{merge} (%)[1, 2]	8.3 (118.0)	19.1 (179.0)	20.2 (179.8)
R_{meas} (%)[1, 4]	8.7 (124.5)	19.8 (185.2)	20.9 (186.1)
R_{pim} (%)[1, 4]	2.7 (41.9)	5.2 (47.5)	5.5 (47.6)
$CC_{1/2}$ [1, 5]	0.997 (0.824)	0.998 (0.711)	0.997 (0.719)
**Refinement**			
Resolution (Å) [1]	28.76-1.30	30.86-2.90	31.27-3.00
Reflections (working/test)[1]	30,537/1,559	138,016/7,326	123,960/6,576
R_{factor} / R_{free} (%)[1,3]	14.5/17.9	17.7/20.0	17.9/20.4
No. of atoms (Protein/Fe^{2+}/sulfate/Mg^{2+} /water)	1,175/2/147	16,106/24/165/-/-	16,144/24/220/37/23
**Model Quality**			
R.m.s deviations			
Bond lengths (Å)	0.007	0.007	0.008
Bond angles (°)	0.922	0.886	0.870
Average B-factor ($Å^2$)			
All Atoms	21.1	63.9	68.9
Protein	19.6	63.4	68.3
Fe^{2+}	17.0	84.1	76.4
sulfate/Mg^{2+}	-	110.1	115.5/66.6
Water	32.8	-	56.8
Coordinate error (maximum likelihood) (Å)	0.13	0.34	0.33
Ramachandran Plot			
Most favored (%)	100	98.2	99.0
Additionally allowed (%)	-	1.6	1.0

1) Values in parenthesis are for the highest resolution shell.

2) $R_{merge} = \Sigma_{hkl}\Sigma_i |I_i(hkl) - \langle I(hkl)\rangle| / \Sigma_{hkl}\Sigma_i I_i(hkl)$, where $I_i(hkl)$ is the intensity measured for the ith reflection and $\langle I(hkl)\rangle$ is the average intensity of all reflections with indices hkl.

3) $R_{\text{factor}} = \Sigma_{hkl} ||F_{\text{obs}}(hkl)| - |F_{\text{calc}}(hkl)|| / \Sigma_{hkl} |F_{\text{obs}}(hkl)|$; Rfree is calculated in an identical manner using 5% of randomly selected reflections that were not included in the refinement.
4) R_{meas} = redundancy-independent (multiplicity-weighted) R_{merge}[1, 2]. R_{pim} = precision-indicating (multiplicity-weighted) R_{merge}[3, 4].
5) $CC_{1/2}$ is the correlation coefficient of the mean intensities between two random half-sets of data [5, 6].

References

1. Evans, P.R., *An introduction to data reduction: space-group determination, scaling and intensity statistics.* Acta Crystallogr D Biol Crystallogr, 2011. **67**(Pt 4): p. 282-92.
2. Evans, P., *Scaling and assessment of data quality.* Acta Crystallogr D Biol Crystallogr, 2006. **62**(Pt 1): p. 72-82.
3. Diederichs, K. and P.A. Karplus, *Improved R-factors for diffraction data analysis in macromolecular crystallography.* Nat Struct Biol, 1997. **4**(4): p. 269-75.
4. Weiss, M.S., *Global indicators of X-ray data quality.* Journal of Applied Crystallography, 2001. **34**: p. 130-135.
5. Karplus, P.A. and K. Diederichs, *Linking crystallographic model and data quality.* Science, 2012. **336**(6084): p. 1030-3.
6. Evans, P., *Biochemistry. Resolving some old problems in protein crystallography.* Science, 2012. **336**(6084): p. 986-7.
